# The glutamate receptor antagonist ifenprodil inhibits hepatitis E virus infection

**DOI:** 10.1128/aac.01035-24

**Published:** 2024-10-03

**Authors:** Mara Klöhn, André Gömer, Qiyu He, Richard J. P. Brown, Daniel Todt, Lin Wang, Eike Steinmann

**Affiliations:** 1 Department of Molecular and Medical Virology, Ruhr University Bochum, Bochum, Germany; 2 Department of Microbiology and Infectious Disease Center, School of Basic Medical Sciences, Peking University Health Science Center, Beijing, China; 3 European Virus Bioinformatics Center (EVBC), Jena, Germany; 4 German Centre for Infection Research (DZIF), External Partner Site, Bochum, Germany; IrsiCaixa Institut de Recerca de la Sida, Barcelona, Spain

**Keywords:** hepatitis E virus, FDA-approved drugs, ifenprodil, NMDA-receptor antagonist

## Abstract

Infection with hepatitis E virus (HEV) represents a global problem, with over 20 million people infected annually. No specific antiviral drugs are available for treating HEV infection, necessitating the development of novel targeted therapeutics. Here, we report that the N-methyl-D-aspartate receptor (NMDAR) antagonist ifenprodil, a clinically approved drug used to treat idiopathic pulmonary fibrosis (IPF), is an HEV inhibitor in liver-derived cells. *In vitro* investigation demonstrates that ifenprodil suppresses viral protein expression in a dose-dependent manner in human hepatoma cells by inhibiting early stages of viral infection. We also found that ifenprodil modulates host cell intrinsic biological processes distinct from virus-induced innate immunity, inhibiting HEV RNA accumulation in primary human hepatocytes. Finally, the inhibitory effect of ifenprodil *in vivo* was also tested in rabbits challenged with the HEV-3ra CHN-BJ-R14 strain. Fecal virus shedding was below the limit of detection in two animals for both ribavirin-treated and ifenprodil-treated rabbits compared to vehicle-treated control animals. Our data demonstrate that ifenprodil is an effective anti-HEV compound with potential as a therapeutic candidate for the treatment of HEV infection.

## INTRODUCTION

Hepatitis E viruses (HEV) is a single-stranded, positive-sense RNA virus with a 7.2 kb genome, belonging to the *Hepevirida*e family (1). HEV infections pose a significant global health burden, causing acute viral hepatitis with varying clinical outcomes ranging from mild, self-limiting disease to fulminant hepatitis. According to statistics, the number of HEV infections globally is as high as 20 million each year, resulting in approximately 44,000–70,000 deaths (2, 3). The HEV burden is particularly pronounced in resource-limited areas, where transmission is facilitated by contaminated water sources and inadequate sanitation conditions. Symptomatic HEV-infected patients generally present with fever, nausea, vomiting, and jaundice (4). In pregnant women, the mortality rate from symptomatic HEV could be as high as 30% (5). Although there is an effective HEV vaccine, it has not yet reached World Health Organization (WHO) prequalification and licensing outside China and Pakistan (6–8). Currently, there are no specifically approved antiviral therapies for the treatment of HEV. Ribavirin (RBV), an approved broad-spectrum nucleoside analog is the only treatment option available; however, its use is hampered by severe side effects, the known emergence of single nucleotide variants and is contraindicated in pregnant women (5, 9–11). Sofosbuvir (SOF), an inhibitor of the HCV RNA-dependent RNA polymerase (RdRp) has been reported as possessing antiviral activity against HEV (12). Although the initial response, as measured by decreasing RNA levels, was promising in recent a pilot study, sustained virological response (SVR) was not achieved (13). Notably, it was demonstrated that the emergence of a substitution from alanine to valine at position 1343 in the viral polymerase was strongly associated with viral relapse (14, 15). Ifenprodil, a phenylethanolamine, which acts as an N-methyl-d-aspartate receptor (NMDAR) antagonist that prevents glutamate signaling, was until recently completed phase 2b clinical trials to treat SARS-CoV-2 infections (16). In addition, the drug has been granted orphan drug designation by the Food and Drug administration (FDA) for the treatment of idiopathic pulmonary fibrosis (IPF) (16). However, its antiviral effect on HEV infection was not previously determined. As part of a drug repurposing strategy, this study explores the anti-HEV activity of ifenprodil both *in vitro* and *in vivo*.

## MATERIALS AND METHODS

### Compounds and drug preparation for animal experiments

For *in vitro* assays, ifenprodil was obtained from MedChemExpress and RBV was purchased from Sigma‐Aldrich. All compounds were stored and diluted according to the manufacturer’s recommendations. For animal testing, Ifenprodil and RBV were purchased from Biochempartner, Shanghai, China.

### Eukaryotic cell culture

Human hepatoma cells (HepG2) were cultivated in Dulbecco’s Modified Eagle Medium (DMEM, ThermoFisher Scientific, Grand Island, NY, USA) enriched with 10% fetal calf serum (FCS), 5% Penicillin/Streptomycin, 5% non-essential amino acids, and 5% L-Glutamine. The HepG2/C3A subclone was grown in Eagle’s Minimum Essential Medium (MEM with glutamine, Invitrogen) supplemented with 10% ultra-low IgG FCS (Invitrogen), 2 mM L-glutamine, 100 µg/mL gentamicin, 1 mM sodium pyruvate, and non-essential amino acids (Gibco). All cells were cultivated on type I collagen-coated plasticware and maintained at 37°C in a 5% CO_2_ incubator. Primary human hepatocytes (PHH) were procured as cryopreserved cells from Primacyt (Schwerin, Germany), thawed according to the manufacturer’s guidelines, and cultured in 24-well plates using Human Hepatocyte Maintenance Medium (HHMM, Primacyt). Donors were confirmed serologically negative for HIV, hepatitis B, hepatitis C, and SARS-CoV-2. Primacyt obtained patient-informed consent, as detailed on its company website.

### HEV constructs and *in vitro* transcription

Human-derived full-length HEV-3 Kernow-C1 (GenBank accession no. JQ679013) virus vector and wild-boar-derived HEV83-2-27 (HEV-3; GenBank accession no. AB740232) virus vector were used to produce full-length cell-culture derived HEV virus particles. Additionally, a construct containing a subgenomic HEV sequence fused with a Gaussia luciferase reporter gene derived from the Kernow-C1 p6 was utilized for replication assays. According to Meister et al. and Todt et al*.* (17, 18), linearized HEV-3 constructs were *in vitro* transcribed, capped with Capping mix and used for transfection into HepG2 cells by electroporation for either virus production or replication assay.

### HEV transfection

For transfection, cells were electroporated following protocols established in previous studies (17). Specifically, a mixture of 5 × 10^6^ cells/mL in 400 µL of Cytomix, supplemented with 2 mM adenosine triphosphate and 5 mM glutathione, was prepared. HepG2 cells were then electroporated with 5 µg of the *in vitro* transcribed HEV RNA using a Gene Pulser system (Bio-Rad, Munich, Germany) and promptly transferred to 10–12 mL of DMEM. Subsequently, cell suspensions were either seeded into 10 cm dishes (10 mL per dish for electroporation with full-length HEV RNA) or into 96-well plates (2 × 10^4^ cells/well for electroporation with subgenomic HEV RNA).

### HEV replication assay

Four hours post-transfection (p.t.) of subgenomic HEV RNA, 50 µL medium, containing the compounds at predetermined concentrations, was added. At various time points p.t. (24, 48, and 96 h), supernatant was collected. Gaussia luciferase activity was quantified by dispensing 20 µL of the harvested cell culture supernatant into each well of a 96-well LUMITRAC 600 plate. This was followed by the addition of coelenterazine substrate. Luminescence was then detected using a Centro XS3 LB 960 luminometer (Berthold Technologies, Bad Wildbad, Germany). The microplate reader was programmed to dispense 50 µL of the substrate into each well; then it was shaken for 2 s before reading the luminescence for a duration of 5 s. Each experimental condition was conducted in triplicates.

### Production of cell culture-derived HEV particles (HEVcc)

Virus stock of HEVcc p6-FL and 83-2-27 was produced as described in HepG2 in DMEM complete with three freeze-thaw cycles in DMEM complete. Capped full-length HEV-3 Kernow-C1 (p6-FL) and HEV83-2-27 *in vitro* transcripts were used for transfection into HepG2 cells by electroporation. Produced virus was harvested and used for titration in HepG2/C3A cells. Subsequently, viral titers were determined by immunofluorescent staining with ORF2 antibody and by counting foci forming units (FFUs/mL). One FFU is defined as one or more ORF2-positive cells separated from another FFU by at least three negative cells.

### HEV infection assays

For infection assays with full-length viruses, HepG2 or HepG2/C3A cells were seeded at a density of 1.5 × 10^4^ cell/well onto collagen-coated 96-well microtiter plates and treated with 100–0.039 µM of compounds 6 h after cell seeding. Cells were then infected with p6-FL virus at a multiplicity of infection (MOI) of 0.1–0.2. Four days post-infection (p.i.) cells were fixated and used for immunofluorescence imaging.

For combination treatment with ifenprodil and RBV, neHEV_CC_ virus-infected HepG2/C3A cells were grown in the presence of various doses of silvestrol or RBV or their combinations. Compounds were mixed at concentrations Ifenprodil:RBV 1:250–1:32,000 and analyzed using SynergyFinder software programmed with R (19). According to the zero interaction potency (ZIP) algorithm, synergy scores were calculated. The ZIP score stands for the response beyond expectation in percentage. In the range of −10 < ZIP < 10, the compounds are likely to act in an additive manner, while a score ≥ 10 indicates synergism, and <−10 shows antagonism.

PHH were inoculated with Kernow-C1 p6 virus (MOI 1) one after cell seeding. Cells were simultaneously treated with either 50, 25, 12.5 µM Ifenprodil, 25 µM RBV, or equal amounts of dimethyl sulfoxide (DMSO). Medium was changed with medium supplemented with drugs on day 1 p.i. Cells were fixed 72 h p.i. for immunofluorescence staining.

### Time-of-addition drug assay

HepG2/C3A cells were seeded at a density of 1.5  ×  10^4^ cells per well in a 96-well plate. The following day, cells were infected with HEV-3-p6-FL (MOI of 0.1–0.2) in assay medium and treated with Ipatasertib (25 µM), K11777 (0.1 µM), RBV (25 µM), or DMSO 1 h before infection (−1 h), the time point of virus infection (0 h) or at 2, 4, 8, 12, 24, 48, and 72 h after infection. At 8 h p.i., the inoculum was removed, and cells were washed thrice with 1× phosphate-buffered saline (PBS). The cells were then incubated with either drug-containing or drug-free medium. Viral infection was monitored 96 h p.i. by indirect immunofluorescence staining against ORF2 capsid protein.

### Cell viability assay

To assess cell viability in hepatoma cells, 0.5 mg/mL of 3-(4,5-dimethylthiazol-2-yl)−2,5-diphenyltetrazolium bromide (MTT) (Sigma) was added to the cells, and the cells were incubated at 37°C in a 5% CO_2_ atmosphere for 1–2 h. After incubation, the medium was discarded, and DMSO was applied to each well. We measured the absorbance at 570 nm for each well using a Tecan microplate absorbance reader. Cells treated with 70% ethanol for 10 min were used as a background control.

For PHH, cytotoxicity was measured using the CytoTox 96 Non-Radioactive Cytotoxicity Assay (Promega). Briefly, 50 µL of supernatant was mixed with an equal volume of CytoTox 96 reagent and allowed to incubate for 30 min at room temperature (RT), shielded from light. Absorbance was then recorded at 492 nm using a Tecan microplate absorbance reader.

### Indirect immunofluorescence

To perform indirect immunofluorescence staining, cells were fixed using 3% paraformaldehyde (PFA) and permeabilized with 0.2% Triton X-100. To prevent any non-specific binding of antibodies, a 5% (v/v) horse serum was used to block the samples for an hour at RT. Next, the cells were stained using an ORF2-specific rabbit hyperimmune serum, which was diluted to 1:5,000 (kindly provided by R. Ulrich from Friedrich Loeffler Institute, Germany). A secondary antibody, goat anti-rabbit (AlexaFluor 488, Life Technologies, Darmstadt, Germany), was then used, which was also diluted in 5% (v/v) horse serum at a dilution of 1:1,000. Finally, the nuclei were stained with 4′,6′-diamidino-2-phenylindole (DAPI; Life Technologies), which was diluted to 1:10,000. Images were taken with a standard wide-field fluorescence microscope (Keyence BZ-X800E) with a 4 × 0.75-numerical aperture (NA) air-objective. DAPI (358 nm) and ORF2 (488 nm) signals were acquired sequentially by using the BZ-X Filter DAPI and BZ-X Filter GFP, respectively.

### RNA sequencing and transcriptome analysis

For transcriptional profiling of HEV-infected and ifenprodil-treated PHH, PHH were seeded on a 24-well plate. One day after cell seeding, cells were inoculated with Kernow-C1 p6 virus (MOI 1) or an equal volume of DMEM complete cell culture medium. Virus inoculum and cell culture medium were supplemented with either 25 µM Ifenprodil, 25 µM RBV, or equal amounts of DMSO (vehicle control). After 24 h of inoculation, the inoculum was removed, and the cells were replenished with 500 µL of fresh cell culture media. After 48 h p.i., cell culture supernatant was completely removed, and the cells were washed once with PBS. Cells were lysed in 350 µL of RA1 buffer of the NucleoSpin RNA kit (Macherey-Nagel) supplemented with 1% beta-mercaptoethanol followed by passing ten times through a narrow-bore syringe (Omican-F, 1 mL, 0.3 × 12 mm, 9161502, B Braun). Total RNA was then extracted using the NucleoSpin RNA kit (Macherey-Nagel) according to manufacturer’s instructions. RNA quality and quantity were analyzed using the NanoDrop One (Thermo Scientific), and sequencing libraries were generated using a NEBNext Ultra II Directional RNA Library Prep Kit (New England Biolabs) according to the manufacturer’s instructions. The resulting libraries were sequenced using the NextSeq 550 platform (Illumina) with single-end 1 × 86 bp setting. For transcriptomic analyses, CLC Genomics Workbench (Qiagen, Aarhaus) was used.

### Quantification of ORF2 positive cells and cells per focus

Mean fluorescence intensities of ORF2 immunofluorescence were obtained from a 5-pixel wide cytoplasm ring following the segmentation of DAPI-stained nuclei using CellProfiler (20). To distinguish noninfected cells from infected cells, a minimum mean intensity threshold was applied. To determine the focus-forming units, individual foci were counted by hand in FIJI (21) using ORF2 immunofluorescence images.

### Western blot analysis

For western blot analysis, HepG2/C3A cells were seeded at a density of 1.9 × 10^5^. By the next day, the cells were infected (MOI 0.1) with HEV-3 Kernow-C1 p6-FL virus and treated with either 25 µM RBV or 25 µM ifenprodil for 4 days. Protein samples were harvested in M-PER buffer (Thermo Scientific, Cat. Nr. 78501) supplemented with 1× Pierce Protease Inhibitor Mix (Thermo Scientific, Cat. Nr. A32953) and spin down at 14,000 *g* for 15 min. To determine the protein concentration, supernatant was used for Pierce Bradford Protein Assay Kit (Thermo Scientific) according to manufacturer’s instructions. A total of 1.35 µg protein was loaded onto gels and run at 100 V for 2 h. Protein was transferred onto nitrocellulose membrane for 2 h at 110 V by wet tank electroblotting. Membranes were blocked with 5% milk in PBS containing 0.05% Tween (PBS-T) and stained with primary rabbit polyclonal antibody anti-ORF2 (#2101; 1:5,000 in 0.5% milk dissolved in PBS-T) over night at 4°C. The next day, membranes were incubated with secondary horseradish peroxidase- (HRP) conjugated polyclonal goat anti-rabbit antibody (Abcam, Cat. Nr. #ab97051, 1:10,000) and β-actin (Sigma-Aldrich, Cat. Nr. A3854, 1:10,000 in 0.5% milk dissolved in PBS-T) for up to 2 h at RT. Protein bands were developed by Pierce ECL Western Blotting Substrate (Thermo Scientific, 32109) on a chemiluminescence membrane imager (Celvin S 420, Biostep Sarstedt).

### Animals and virus strains used in *in vivo* experiments

Fifteen Japanese white rabbits, aged about three months weighing between 2.8 and 3.0 kg, were randomly chosen and obtained from the Department of Laboratory Animal Science at the Peking University Health Science Center. All rabbits were kept in independent cages, and adequate clean water and food were given. Before inoculation, all animals were confirmed to be HEV RNA negative in collected feces samples by real-time quantitative PCR (RT-qPCR). For the inoculation of rabbits, the HEV-3ra strain (CHN-BJ-R14, genotype 3, GenBank JX109834), which was recovered from a fecal samples of a farmed rabbit in Beijing, was used.

### Ifenprodil treatment in rabbits

Fifteen Japanese white rabbits were randomly assigned into three groups with five rabbits each. Animals were inoculated with 1 mL HEV-3ra inoculum intravenously. At 2 weeks post-inoculation drugs were administrated daily with 10 mg/kg/day (Ifenprodil) or 33.3 mg/kg/day. As a vehicle control, five rabbits were administrated with DMSO/water and remained untreated throughout the whole experiment.

### Extraction and detection of HEV RNA and HEV antigen in rabbit feces

Viral RNA was extracted from 200 µL fecal suspensions or serum samples using EasyPure Viral DNA/RNA Kit (TransGen Biotech, Beijing, China) according to manusfractureres instructions. HEV RNA in all fecal samples was quantified by RT-qPCR with GoTaq Probe 1-step RT-qPCR System (Promega Corporation, Wisconsin, USA).

### Software and data analysis

Dose-dependent inhibition of infection was plotted and adjusted to a non-linear regression model using GraphPad Prism v10.1.2 for Windows (La Jolla, California, USA, www.graphpad.com). EC_50_ and CC_50_ were calculated using the four-parameter log-logistic model, and a statistical analysis was performed. Illustrations were generated with Adobe Illustrator.

## RESULTS

**FIG 1:**
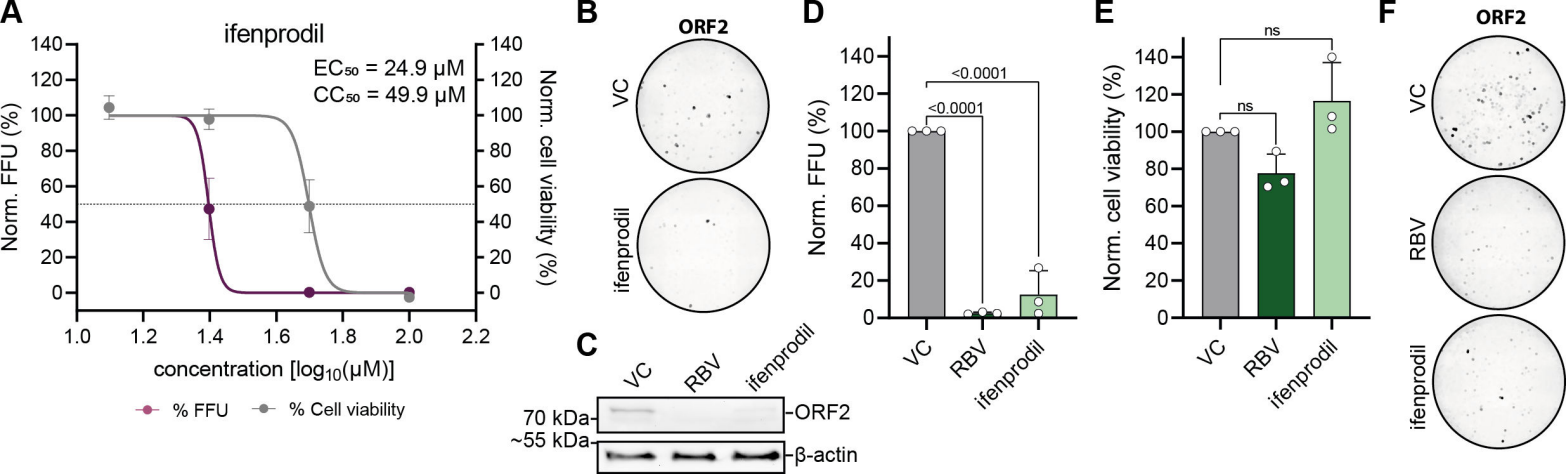
Antiviral activity of ifenprodil against HEV in human hepatoma cells. (A) Dose-dependent non-enveloped HEV (neHEV; Kernow-C1/p6) inhibition by ifenprodil on intracellular HEV ORF2 expression in HepG2 cells (*n* = 3). Normalized infections in percent (%) were fitted by four-parameter log-logistic model to determine half-maximum EC_50_ and half-maximum CC_50_. (B) Representative whole well images of neHEV infected cells treated with either vehicle control (DMSO) or 50 µM ifenprodil. (C) Western blot analysis (*n* = 2) of HEV-3 Kernow-C1 p6-FL infected HepG2/C3As treated with 25 µM ifenprodil. Treatment with 25 µM RBV and DMSO served as positive and negative control, respectively. (D) Antiviral effect and (E) cytotoxic effects of 20 µM ifenprodil on wild boar neHEV 83-2-27 virus. Statistical significance was determined by an ordinary one-way ANOVA and corrected for multiple comparison by Dunnett. *P*-values < 0.05 were considered significant. (F) Representative immunofluorescence images illustrate the impact of ifenprodil on the infectivity of neHEV.

We first evaluated the antiviral activity of ifenprodil in cell culture using human hepatoma cells. HepG2 cells were inoculated with the non-enveloped (ne) HEV-3 Kernow-C1 p6 virus in the absence or presence of serial dilutions of ifenprodil (Fig. 1A). HEV infection rates were evaluated by detecting ORF2 capsid protein expression via fluorescence microscopy. Treatment with DMSO and RBV served as positive and negative controls, respectively. Four days p.i., ORF2 expression decreased with a half-maximum effective concentration (EC_50_) of 24.9 µM and a half-maximum cytotoxic concentration (CC_50_) of 49.9 µM (Fig. 1A and B). The inhibitory activity of ifenprodil on ORF2 expression was also confirmed by western blotting of ifenprodil-treated HEV-3 Kernow-C1 p6-FL virus-infected HepG2/C3A cells (Fig. 1C). Furthermore, we observed a significant reduction in infection rates in 83-2-27 infected cells upon ifenprodil treatment (Fig. 1D and F), without a major effect on cell viability (Fig. 1E).

**FIG 2:**
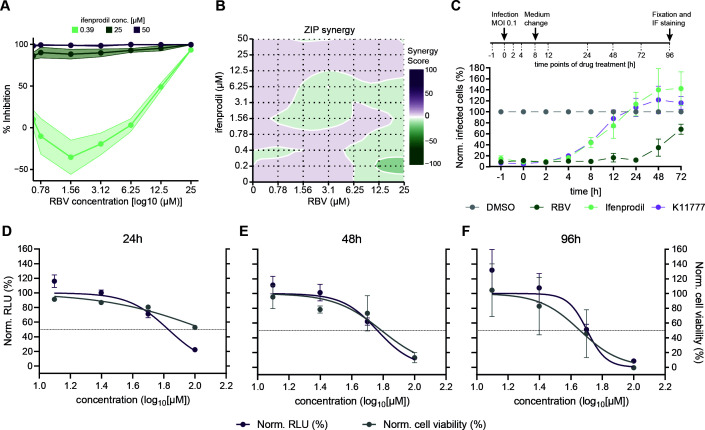
Combination treatment with RBV and time-of-drug addition analysis. (A) *In vitro* effect of antiviral combinations on HEV infection in HepG2/C3A cells. HEV inhibition during treatment with 0.39 µM (light green), 25 µM (dark green) or 50 µM (purple) ifenprodil and simultaneous titration of RBV (from 0 to 25 µM). (B) A two-dimensional map of synergy scores shown for the combination of ifenprodil with RBV. Synergy scores were based on ZIP synergy analysis determined with SynergyFinder (19). Determined ZIP score was −0.37. The ZIP score stands for the response beyond expectation in percentage. In the range of −10 < ZIP < 10, the compounds are likely to act in an additive manner, while a score ≥10 indicates synergism, and <−10 shows antagonism. (C, top) Experimental setup of the time-of-drug-addition assay. Hepatoma HepG2/C3A cells were inoculated with neHEV at 0 h and treated with DMSO, 0.1 µM K11777, or 25 µM RBV for 1 h (−1) before, during (0), and 2, 4, 8, 12, 24, 48, and 72 h after infection until fixation at 96 h p.i. At 8 h after infection, inoculum was removed and replenished with fresh medium-containing drugs after several washes with PBS. (C, bottom) The inhibitory effect of 20 µM ifenprodil on neHEV infection when added at different time points pre- or post-infection is depicted by the green curve. The broad-spectrum RNA virus inhibitor RBV (25 µM) served as positive control (dark green curve). Data presented represent mean  ±  SD from three independent experiments. (D–F) Hepatoma cells were transfected with HEV-3 Kernow-C1 p6-Gluc subgenomic replicon and treated with RBV (100–12.5 µM; 1:2 serial dilution) or ifenprodil (100–12.5 µM; 1:2 serial dilution). Depicted are normalized relative light units (RLUs) measured 24 (D), 48 (E) and 72 h (F) post-electroporation. DMSO was employed as vehicle control. The depicted values represent means ± SD from two independent experiments.

Given that the combination of multiple antiviral drugs is a common strategy to enhance antiviral efficacy, limit toxicity, and avoid drug resistance, we next evaluated potential synergies between ifenprodil and RBV. With a ZIP synergy score of −0.37 (<−10 = antagonistic; from −10 to 10 = additive; >10 = synergistic), ifenprodil was observed to have notable levels of additive antiviral effect in combination with RBV (Fig. 2A and B). However, these data suggest a combinatorial approach should be further investigated to determine whether *in vivo* and clinical synergism or antagonism exist between the two drugs.

We next evaluated whether ifenprodil acts on entry or post-entry steps of the viral life cycle. We first conducted time-of-addition studies, which compared the effects of the compound administered prior (−1), concurrently with (0 h), or 2, 4, 8, 12, 24, 48, and 72 h after viral challenge (Fig. 2C). We included the treatment with K11777 (22), a previously described HEV entry inhibitor, and RBV, a known HEV replication inhibitor into our analysis. When introduced to cultures before infection as well as 4 h p.i., ifenprodil (similar to K11777) retained its antiviral efficacy (Fig. 2C). However, a time-dependent loss in antiviral activity was observed when ifenprodil was administered for or beyond 8 h p.i., suggesting that ifenprodil potentially targets the early onset of the viral replication cycle. In contrast, HEV infection was suppressed only when RBV was added after the onset of RNA virus replication (~24–72 h p.i.). In accordance with these results, when tested in a subgenomic reporter assay, ifenprodil did not inhibit HEV RNA replication 24, 48, or 72 h p.t. (Fig. 2D through F).

**FIG 3:**
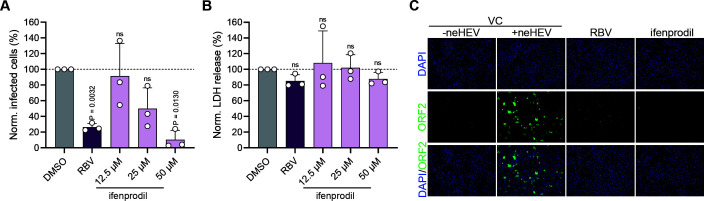
Antiviral activity of ifenprodil against HEV in PHH. (A) The effects of ifenprodil on intracellular ORF2 expression in PHH (*n* = 3). PHH were infected with Kernow-C1/p6 virus for 3 days and treated with 12.5, 25, and 50 µM ifenprodil. Treatment with DMSO and 25 µM RBV served as negative and positive controls, respectively. (B) Cell viability was determined by LDH release assay. Data depict means + SD of three independent experiments. To test the significance of mean differences, one-way ANOVA, followed by Dunnett multiple comparison test, was used. *P*-values > 0.05 were considered to be not significant (ns). (C) Representative immunofluorescence images of PHH. ORF2 = green; DAPI = blue.

PHH represent an authentic model for HEV infection and antiviral drug testing. Therefore, we validated the antiviral activity of ifenprodil against HEV in PHH. Treatment with increasing doses of ifenprodil resulted in markedly reduced viral infection compared with vehicle-treated cells (Fig. 3A and C), while cell viability was not affected by treatment with ifenprodil (Fig. 3B and C).

**FIG 4:**
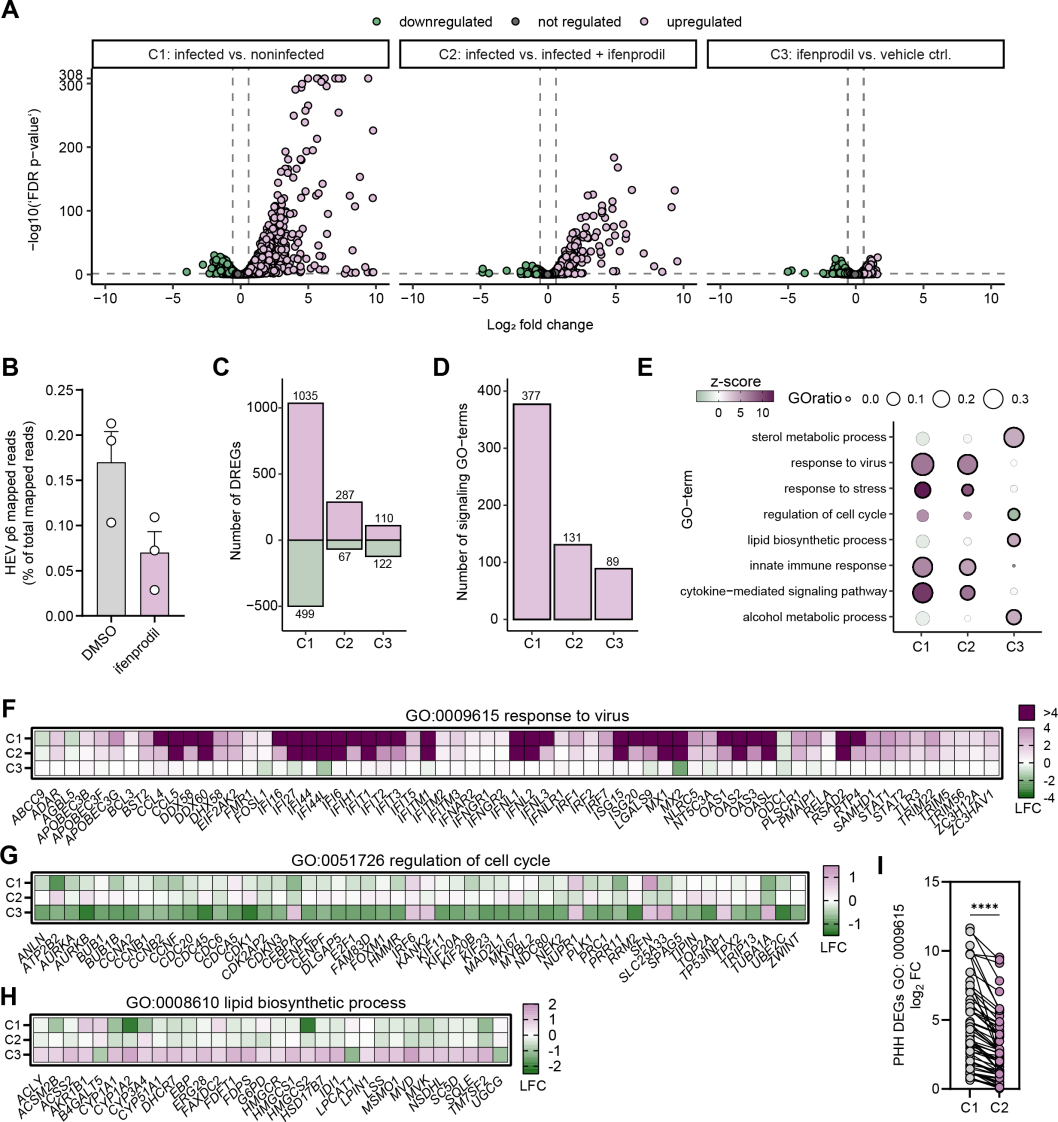
Distinct host transcriptional responses upon HEV infection and ifenprodil treatment in PHH. (A) PHH from three different donors were either inoculated with HEV-3 Kernow-C1 p6 virus or with DMEM and/or treated with 25 µM ifenprodil. Total RNA was extracted 48 h p.i., and Illumina sequencing was performed before mapping to human (Hg38) or HEV (Kernow-C1 p6) genome scaffolds. Volcano plots visualize DEGs induced in infected versus uninfected cells (condition 1; C1), for ifenprodil versus vehicle control (DMSO) treated cells (condition 2; C2), and in infected + ifenprodil treated versus infected + vehicle treated cells (condition 3; C3). (B) Percentage of HEV p6 mapped reads of total mapped reads, under DMSO and ifenprodil treatment. (C) Numbers of upregulated and downregulated DEGs (reads per kilobase million [RPKM] ≥ 0.5 and fold change ≥ 2) under the indicated conditions. Purple bars indicate upregulated genes, and green bars represent downregulated genes. (D) Number of significantly enriched GO-terms activated in condition 1, 2, and 3 (false discovery rate [FDR] ≤ 0.05). (E) Dot plot visualizes representative enriched GO categories under the indicated conditions. Circle size corresponds to the size ratio of enriched gene relative to total genes in that category, color to the z-score, and border to significance (black border = FDR ≤ 0.05; no border = ns). (F–H) Heat maps visualizing dysregulation of genes (mean log_2_ FC) associated with the GO category “response to virus” (GO:0009615; panel F), “regulation of cell cycle” (GO0051726; panel G) “lipid biosynthesis process” (GO0008610; panel H) under the indicated conditions. Color corresponds to log_2_-fold change (LFC). (I) Comparison of the mean log_2_ FC of genes clustering in the GO category “response to virus” upon infection of PHH under the indicated conditions. Statistical significance was calculated using two-tailed, paired *t*-test, ****P < 0.0001. Data presented derived from infection *n* = 3 biological replicates. FC, fold change.

Next, we compared host transcriptional responses in vehicle control-treated (DMSO) PHH to HEV-infected PHH (neHEVCC, Kernow-C1/p6), PHH treated with ifenprodil (20 µM), or PHH where infection and treatment were combined. PHH were derived from three different donors, and total RNA was extracted at 48 h p.i. for subsequent RNA-Sequencing. Analysis of differentially expressed genes (DEGs) identified distinct host responses apparent between conditions (Fig. 4A and C). Of note, compared to untreated cells, HEV transcript levels were ~60% reduced in cells treated with ifenprodil (Fig. 4B). Furthermore, gene ontology (GO) analysis identified host biological processes targeted by HEV inoculation and ifenprodil treatment (Fig. 4D through H). GO enrichment analysis of significant DEGs in HEV-infected PHH relative to uninfected cells revealed significant enrichment of pathways related to virus response, innate immune responses, cytokine-mediated signaling pathways, and stress response (Fig. 4E and F). This underscores the ability of PHH to detect HEV infection, initiate interferon release, and activate innate immune responses. Although similar GO categories were enriched in ifenprodil-treated infected cells, the presence of ifenprodil significantly dampened the induction of these pathways compared to HEV infection alone (Fig. 4I). In contrast, in uninfected ifenprodil-treated PHH, pathways including sterol and alcohol metabolic processes, along with the regulation of cell cycle and lipid biosynthetic processes were targeted (Fig. 4E, G and H). Altogether, these analyses identified distinct host responses to HEV infection and ifenprodil treatment and highlight reduced HEV RNA and concomitant innate induction in HEV-infected, ifenprodil-treated cells.

**FIG 5:**
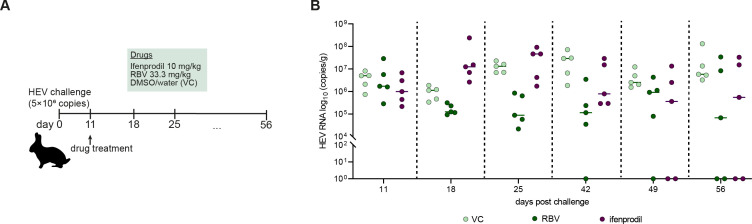
*In vivo* efficacy of ifenprodil against HEV. (A) Schematic representation of the *in vivo* study setup. Twelve-week-old male rabbits were inoculated with 5 × 10^6^ copies HEV RNA by intravenous injection (*n*  =  5 in the vehicle-treated group, *n* = 5 in the compound-treated group). Ifenprodil treatment was given daily by intraperitoneal injection, beginning 14 days p.i. until 31 days p.i. (B) The suppressive effect of ifenprodil on the viral load in the feces on day 11, 18, 25, 42, 49, and 56 after infection. Rabbits received the treatment once daily with RBV 33.3  mg/kg (dark green dots), vehicle (grey dots), or ifenprodil at 10 mg/kg (bright green dots). Individual data points are represented by dots.

To further corroborate the utility of ifenprodil *in vivo*, the drug was tested in HEV-infected rabbits. For a comparative analysis, the experimental groups included rabbits treated once-daily with vehicle (*n* = 5, negative controls), RBV (33.3 mg/kg, *n* = 5, positive controls), or ifenprodil (10 mg/kg, *n* = 5). Treatment started 2 weeks after infection and continued until day 31 p.i. (Fig. 5A). After 49 and 56  days of infection, fecal virus shedding determined by qRT-PCR was not detectible in two animals for both RBV-treated and ifenprodil-treated rabbits compared to vehicle-treated control animals (Fig. 5B), indicating that some but not all tested animals cleared HEV infection.

## DISCUSSION

NMDARs are ligand-gated ion channels and ionotropic glutamate receptors, which are involved in excitatory neurotransmission, neuronal development, and synaptic plasticity upon binding glycine and glutamate (23). Interestingly, NMDARs have also been found to regulate virus-induced neuronal cell damage for a variety of viruses, including Japanese encephalitis virus (24). To that end, several NMDA receptor antagonists have been identified to prevent, protect, or reverse virus-induced neurodegeneration of various viruses including measles virus, HIV, Zika virus (ZIKV), and Sindbis virus (25–28). Specifically, studies have demonstrated that ifenprodil can reverse synapse loss and cogitative impairment produced by the HIV-1 Protein Tat *in vivo* and prevent neuronal death induced by the ZIKV without inhibiting virus replication (26, 27). In contrast, our study demonstrated that ifenprodil significantly reduces HEV-3 Kernow-C1/p6 and HEV-3 83-2-27 infections in HEV-infected hepatoma cells and decreases the infectivity of the HEV-3 Kernow-C1/p6 virus in PHH. The fact that NMDAR subunits have previously been found to be absent in liver tissue suggests that its antiviral mechanism in PHH might not directly involve NMDAR inhibition (29). Instead, ifenprodil might also target other cellular factors, as it is also able to antagonize several receptors including α1, 5-HT, and σ1, σ2 receptors (30). Further studies testing other NMDAR blockers, such as dizocilpine (MK-801), agmatine sulfate, or the FDA-approved drug dextromethorphan (Auvelity) would be a strategic approach to validate NMDAR as a potential target in future studies. Furthermore, whether ifenprodil is active against HEV-1 Sar55 remains elusive. Additionally, RNA sequencing analysis showed that the genes impacted by ifenprodil are significantly associated with GO functional terms and pathways involved in the regulation of the cell cycle and lipid biosynthetic processes (31). This finding aligns with observations that NMDARs are implicated in lipid metabolism regulation in the liver (32). Notably, we did not observe any induction of antiviral pathways during ifenprodil treatment in HEV infected cells. Therefore, further studies are necessary to elucidate the precise antiviral mechanisms. Finally, we also observed that fecal virus shedding was not detectible in two out of five rabbits treated with either RBV or ifenprodil (31). However, considering that RBV typically serves as the positive control yet did not show effectiveness in all animals might indicate potential issues with the experimental setup or execution, such as variability in dose administration, animal condition, or other methodological errors. Therefore, future studies need to re-evaluate the antiviral effect of ifenprodil *in vivo* and should explore different concentrations of the drug to refine dosing parameters before clinical trials can assess its efficacy for treating HEV. Additionally, screening structural derivatives of ifenprodil could enhance antiviral activity, offering potential improvements over the original molecule (33). Interestingly, based on the observations that ifenprodil reduces acute lung injury in a murine model infected with the H5N1 influenza virus, Algernon Pharmaceuticals assessed the safety and efficacy of ifenprodil in treating COVID-19 in a phase 2b/3 trial (NCT04382924). Despite completing the study in 2023, Algernon Pharmaceuticals decided not to proceed with a phase 3 clinical trial for COVID-19 treatment using ifenprodil, a decision that was influenced by the widespread global distribution of COVID-19 vaccines, the high costs associated with advancing to phase 3, and the availability of various other therapeutic options that have been authorized (16, 34).

## Data Availability

RNA-seq data generated in this study and subsequent downstream analyses, including identification of DEGs, enriched GOs categories, and targeted pathways, have been deposited in the NCBI GEO database under accession number GSE274780.
